# Association between intensity and type of physical activity and lung function in patients with COPD: a population-based study

**DOI:** 10.3389/fmed.2026.1878202

**Published:** 2026-07-24

**Authors:** Eunjoo Lee, Kyungdo Han, Sun Jae Won, So-Youn Chang

**Affiliations:** 1 Department of Rehabilitation Medicine, College of Medicine, Yeouido St. Mary’s Hospital, The Catholic University of Korea, Seoul, Republic of Korea; 2 Department of Statistics and Actuarial Science, Soongsil University, Seoul, Republic of Korea

**Keywords:** aerobic exercise, COPD, lung function, physical activity, population-based study

## Abstract

**Introduction:**

Aerobic exercise is a cornerstone of chronic obstructive pulmonary disease (COPD) management; however, its association with lung function parameters has remained inconsistent across prior studies, and previous studies have been limited by examining physical activity (PA) as a single intensity dimension without distinguishing specific PA types. We therefore investigated the associations between PA intensity and type with lung function outcomes (FEV1, FVC, and PEF) in COPD patients and individuals with normal spirometry (FEV1/FVC ≥ 0.7 and FVC ≥ 80% of predicted), and formally tested whether these associations differ between the two groups.

**Methods:**

This nationwide population-based cross-sectional study included data from 14,679 participants from 2014 to 2019. All participants underwent a health survey and a series of health examinations, including a pulmonary function test. Associations between lung function and PA intensity, PA types (walking, strengthening, and aerobic exercise), and PA intensity stratified by age and sex were assessed using analysis of covariance.

**Results:**

In the COPD group, higher levels of PA were associated with increased forced expiratory volume in one second (FEV1), forced vital capacity (FVC), and peak expiratory flow (PEF) across all models. Among PA types, aerobic exercise was the only type significantly associated with improved FEV1, FVC, and PEF in COPD patients across progressively adjusted models (unadjusted; adjusted for sociodemographic and lifestyle factors; additionally adjusted for major comorbidities). Furthermore, the associations between aerobic exercise and FEV1 and FVC were significantly stronger in the COPD group than in the normal spirometry group in adjusted analyses (*p* for interaction = 0.0297 and 0.0069, respectively, in the fully adjusted model), whereas no significant interaction was observed in the unadjusted model. No significant associations between PA intensity and lung function were observed in COPD patients when stratified by age or sex.

**Discussion:**

Among PA types, aerobic exercise was the only type significantly associated with improved FEV1, FVC, and PEF in COPD patients after full covariate adjustment, and its associations with FEV1 and FVC were significantly stronger in the COPD group than in individuals with normal lung function. These findings support the inclusion of structured aerobic exercise as a core component of COPD management.

## 1 Introduction

Chronic obstructive pulmonary disease (COPD) is a heterogeneous lung condition caused by abnormalities of the airways (bronchitis, bronchiolitis) and/or alveoli (emphysema), leading to chronic respiratory symptoms such as dyspnea, cough, and sputum production, and is associated with episodes of acute exacerbation (1). This disease is a major cause of morbidity, mortality, and healthcare utilization worldwide (2). Recent estimates suggest that approximately 328 million individuals globally are affected by COPD (3). The prevalence of COPD continues to increase, driven primarily by aging populations and persistent exposure to risk factors such as smoking, air pollution, and occupational hazards (4).

Many patients with COPD have reduced levels of physical activity (PA) because of symptoms such as breathlessness and fatigue, which further contributes to the decline in their physical condition and lung function (5). A recent meta-analysis of 11 studies involving 900 COPD patients demonstrated that physically active participants had better emotional wellbeing, less fatigue, improvements in health-related quality of life, muscle strength, exercise capacity, and reduced dyspnea (6). Furthermore, PA has been associated with attenuated disease progression and reduced risk of exacerbations and mortality in COPD (7, 8). Collectively, these findings highlight the critical role of PA in the management and prognosis of COPD.

Previous research has demonstrated a positive association between PA and lung function in patients with COPD, including evidence that higher PA attenuates longitudinal decline in FEV1 and FVC (7, 9). However, only a limited number of studies have explored this relationship, and most have categorized PA by intensity rather than by type. Critically, no large-scale population-based study has investigated whether the association between specific PA types and lung function differs between patients with COPD and those with normal lung function. The present study aimed to address these gaps by examining the associations of both PA intensity and PA type with lung function in a nationally representative sample, categorizing PA into walking, strengthening exercise, and aerobic exercise, and formally testing whether these associations differ between COPD patients and individuals with normal lung function. We also performed subgroup analyses stratified by age and sex to assess potential effect modification.

## 2 Materials and methods

### 2.1 Study population

This nationwide population-based cross-sectional study investigated data from the Korea National Health and Nutrition Examination Survey (KNHANES), a national surveillance system between 2014 and 2019. The Korea Centers for Disease Control and Prevention (KCDC) has annually conducted the KNHANES since 1998. The KNHANES collects nutritional and health data from the population of the Republic of Korea (hereafter “Korea”). The survey integrates them using a multi-stage, stratified, and clustered probability sampling method to ensure national representativeness (10). Participants were eligible if they were aged 40 years or older, had completed spirometry with an obstructive or normal ventilatory pattern, completed the PA questionnaire (GPAQ), and provided complete data on all covariates including sociodemographic factors, anthropometric measures, and comorbidities. Of the 27,393 participants initially assessed, those with missing spirometry data, a restrictive ventilatory pattern, missing PA data, or missing covariate data were excluded, resulting in a final analytic sample of 14,679 participants. The participant selection process is illustrated in Supplementary Figure 1, and the inclusion and exclusion criteria are summarized in Supplementary Table 2.

### 2.2 Ethics approval and informed consent

This study was approved by the Institutional Review Board of the KCDC, and all participants provided written informed consent. Our institution’s IRB granted an exemption from ethical approval for this study based on its use of publicly accessible data.

### 2.3 Demographic and clinical characteristics

The KNHANES evaluates the health and nutritional conditions of participants through a structured health interview, health examination, and nutrition survey. All procedures were conducted in person by trained medical personnel in strict adherence to standardized protocols. All equipment was regularly calibrated to ensure accuracy (10). This study analyzed participants’ demographic and clinical characteristics, which comprised sociodemographic and anthropometric factors, comorbidities, and clinical and laboratory data.

The analyzed sociodemographic factors were age, sex, income, education categorized by a 12-year education period, smoking status, and drinking level. Income tiers were divided into quartiles according to the national median household income, with the lowest quartile defined as low income. Participants were categorized into non-smokers, former smokers, and current smokers. Non-smokers were individuals who had smoked fewer than five cigarettes in their lifetime, while former smokers were those with a history of smoking more than five cigarettes but who had subsequently quit. Heavy drinking, as defined by the Korean Ministry of Health and Welfare, refers to the consumption of ≥7 standard drinks for men and ≥5 for women on at least 2 days per week, whereas mild drinking is defined as consumption of up to four drinks per day for men and two for women.

Dynapenia is a loss in muscle strength associated with aging and is caused by multifactorial biological contributors, not neurologic or muscular diseases (11). Handgrip strength is considered a measure of muscle strength and is defined as <26 kg for men and <18 kg for women by the Asian Working Group for Sarcopenia criteria (12). Other anthropometric measures included body mass index (BMI) and waist circumference (WC). Comorbidities, including hypertension, diabetes mellitus (DM), dyslipidemia, asthma, cerebrovascular disease (CVD), and depression, were assessed based on participant self-reports obtained through the standardized health interview questionnaires of the KNHANES, which are administered by trained interviewers in accordance with protocols established by the Korea Disease Control and Prevention Agency (KDCA) and have been validated for use in large-scale national health surveys (10). Among the laboratory data, fasting serum glucose, total cholesterol, high-density lipoprotein cholesterol (HDL-C), and triglycerides were analyzed.

### 2.4 Measurement of lung function

All participants in this study underwent a pulmonary function test (PFT), including measurements of FEV1, FVC, and PEF using spirometry. This test was performed by qualified medical technicians in adherence to the guidelines established by the American Thoracic Society/European Respiratory Society Task Force. According to the Global Initiative for Chronic Obstructive Lung Disease (GOLD) strategy reports, the diagnosis of COPD is established by spirometry, requiring a post-bronchodilator FEV1/FVC ratio < 0.7 and disease severity is determined primarily based on FEV1 measurements (13). Normal pulmonary function was characterized by both a preserved FEV1/FVC ratio and an FVC of 80% or greater. Based on these criteria, participants were classified into the COPD and normal spirometry groups (hereafter referred to as the “normal group”).

### 2.5 Assessment of physical activity

Physical activity was assessed using the Korean Global Physical Activity Questionnaire (GPAQ), a validated instrument that captures the weekly frequency and duration of activities across three domains: work-related activity, travel (e.g., walking or cycling for transport), and recreational activity (14). Based on WHO guidelines, participants were classified into three PA intensity levels — low, moderate, and high — according to the total volume of activity in MET-minutes per week (15, 16).

High PA levels were defined as meeting any of the following criteria: engaging in vigorous-intensity activity on at least 3 days per week, accumulating a minimum of 1,500 metabolic equivalent of task (MET)-minutes per week or combining walking, moderate-, and vigorous-intensity activities on seven or more days to achieve 3,000 MET-minutes per week. Moderate PA levels were assigned to participants who engaged in vigorous-intensity activity for at least 20 min on three or more days, participated in moderate-intensity activity or walking for at least 30 min on five or more days, or accumulated at least 600 MET-minutes per week through a combination of activities. Those who did not meet the criteria for either the high or moderate categories were classified as having low PA levels (15).

In addition to PA intensity, we examined adherence to three distinct PA types — walking, strengthening exercise, and aerobic exercise — each representing a different physiological domain: daily ambulatory activity, musculoskeletal function, and cardiopulmonary capacity, respectively. Walking participation was defined as at least 30 min per session on five or more days in the past week. Strengthening exercises include push-ups, sit-ups, dumbbell workouts, weightlifting, or pull-ups, performed on at least 2 days in the past week. Aerobic exercise participation was determined based on meeting a threshold of 150 min of moderate-intensity activity, 75 min of vigorous-intensity activity, or an equivalent combination of both within the past week. Moderate-intensity activities included brisk walking, doubles tennis, or cycling at a regular pace, while vigorous-intensity activities included running, aerobics classes, fast cycling, or hiking.

### 2.6 Statistical analysis

Demographic and clinical values are presented as mean ± standard error (SE). These characteristics across the groups were compared by analyzing continuous variables with the *t*-test and categorical variables with the chi-square test. Using analysis of covariance (ANCOVA), the study evaluated the associations between PA intensity level and lung function, and between adherence to specific PA types and lung function, as well as the relationships of PA intensity level and lung function with age and sex in the normal and COPD groups.

To investigate potential confounding effects, three models were constructed with adjustments for covariates. Model 1 was unadjusted. Model 2 was adjusted for age, sex, BMI, income, education, drinking, and smoking, while Model 3 additionally incorporated adjustments for DM, hypertension, and dyslipidemia. To evaluate whether the association between physical activity and lung function differed by group, the *p*-value for interaction was assessed. All statistical analyses were performed using SAS version 9.4 (SAS Institute Inc., Cary, NC, United States), accounting for the complex sample design. The tests were two-sided with a significance level set at 5%.

## 3 Results

### 3.1 Demographic and clinical characteristics of participants

Table 1 presents the demographic and clinical characteristics of participants of the normal and COPD groups. Compared with normal group, participants with COPD were older, predominantly male, and had a lower level of education. They were also more frequently current or former smokers and heavy drinkers. The COPD group showed a higher proportion of individuals with lower BMI (<18.5 kg/m^2^). Of comorbid conditions, hypertension, diabetes mellitus, asthma, and cardiovascular disease were significantly more prevalent in the COPD group. In addition, HDL-C levels were lower and triglyceride levels were higher in the COPD group. Lung function parameters revealed that participants with COPD had reduced FEV1 and PEF. For physical activity, no significant differences were observed between the two groups across each physical activity intensity level; however, the COPD group demonstrated greater participation in strengthening exercise compared to the normal group.

**TABLE 1:** The demographic and clinical characteristics of participants.

Characteristics	Normal	COPD	*P-*value
	(*n* = 12,386)	(*n* = 2,293)	
Age (years)			<0.0001*
40–49	40.1 (0.63)	11.7 (0.97)	–
50–59	35.3 (0.55)	25.6 (1.23)	–
60–69	17.0 (0.43)	32.8 (1.16)	–
≥70	7.6 (0.27)	29.9 (1.07)	–
Sex			<0.0001*
Male	43.8 (0.46)	74.9 (1.02)	–
Female	56.2 (0.46)	25.1 (1.02)	–
Low income	34.8 (0.78)	23.0 (1.23)	<0.0001*
Education			<0.0001*
Education years < 12	27.0 (0.60)	46.3 (1.35)	–
Education years ≥ 12	73.0 (0.60)	53.7 (1.35)	–
Smoking			<0.0001*
Non-smoker	61.2 (0.51)	31.8 (1.13)	–
Former smoker	21.2 (0.43)	37.3 (1.22)	–
Current smoker	17.6 (0.45)	30.9 (1.21)	–
Drinking			<0.0001*
Non	25.9 (0.47)	27.1 (1.08)	–
Mild	65.6 (0.52)	60.1 (1.23)	–
Heavy	8.5 (0.32)	12.9 (0.83)	–
Dynapenia	5.8(0.27)	8.2 (0.63)	0.0787
BMI level (kg/m^2^)			0.0399*
<18.5	1.8 (0.14)	2.3 (0.36)	–
18.5 ≤ <23	37.5 (0.50)	37.4 (1.18)	–
23 ≤ <25	26.5 (0.45)	26.4 (1.15)	–
25 ≤ <30	30.5 (0.48)	31.5 (1.19)	–
≥30	3.8 (0.20)	2.4 (0.37)	–
Waist circumference	82.7 ± 0.10	85.7 ± 0.21	<0.0001*
Comorbidities			
Hypertension	31.2 (0.52)	44.9 (1.21)	<0.0001*
Diabetes mellitus	12.8 (0.36)	20.8 (0.96)	<0.0001*
Dyslipidemia	24.7 (0.48)	24.2 (1.05)	0.7104
Asthma	1.5 (0.13)	7.2 (0.60)	<0.0001*
CVD	2.8 (0.16)	6.6 (0.53)	<0.0001*
Depression	4.7 (0.21)	3.8 (0.50)	0.1453
Fasting glucose	101.7 ± 0.25	105.6 ± 0.57	<0.0001*
Total cholesterol	198.3 ± 0.40	188.8 ± 0.95	<0.0001*
HDL-C	51.4 ± 0.14	47.9 ± 0.29	<0.0001*
Triglyceride	117.9 (116.3–119.4)	127.8 (124.2–131.4)	0.0207*
FEV1	2.9 ± 0.01	2.3 ± 0.02	<0.0001*
FVC	3.6 ± 0.01	3.7 ± 0.03	0.5964
Peak expiratory flow	7.4 ± 0.02	6.2 ± 0.05	<0.0001*
Physical activity-intensity			0.1861
Low	60.6 (0.54)	62.9 (1.24)	–
Moderate	30.0 (0.48)	27.7 (1.14)	–
High	9.5 (0.33)	9.5 (0.82)	–
Physical activity-type			
Walking	38.9 (0.55)	38.9 (1.22)	0.9614
Strengthening exercise	21.3 (0.44)	25.8 (1.14)	<0.0001*
Aerobic exercise	46.7 (0.56)	44.6 (1.28)	0.1155

*P < 0.05. Values are presented as mean ± standard error (SE), percentage (SE), or geometric mean (95% confidence interval). The *p*-values were analyzed using *t*-test for continuous variables or χ^2^ test for categorical variables. COPD, chronic obstructive pulmonary disease; BMI, body mass index; CVD, cerebrovascular disease; HDL-C, high-density lipoprotein cholesterol; FEV1, forced expiratory volume in one second; FVC, forced vital capacity.

### 3.2 Association between the intensity level of physical activity and lung function

In both the normal and COPD groups, higher levels of physical activity were associated with increased FEV1 and FVC across all models, as shown in Table 2. For PEF, however, this positive association was observed only in the COPD group across all models. The *p*-values for interaction were not statistically significant, indicating that there were no significant differences by group.

**TABLE 2:** Association between intensity level of physical activity and lung function.

Variables	Group	Physical activity intensity level	Model 1^†^	Model 2^‡^	Model 3^§^
FEV1	Normal	Low	2.84 ± 0.01	2.81 ± 0.01	2.80 ± 0.01
		Moderate	2.88 ± 0.01	2.82 ± 0.01	2.81 ± 0.01
		High	3.14 ± 0.02	2.86 ± 0.01	2.85 ± 0.01
		*P*-value	<0.0001*	0.0038*	0.0044*
	COPD	Low	2.29 ± 0.02	2.24 ± 0.02	2.23 ± 0.02
		Moderate	2.38 ± 0.03	2.31 ± 0.03	2.29 ± 0.03
		High	2.57 ± 0.06	2.31 ± 0.04	2.29 ± 0.04
		*P*-value	<0.0001*	0.0258*	0.0302*
	*P*-value for interaction		0.3132	0.1407	0.1479
FVC	Normal	Low	3.58 ± 0.01	3.59 ± 0.01	3.57 ± 0.01
		Moderate	3.63 ± 0.02	3.60 ± 0.01	3.58 ± 0.01
		High	3.98 ± 0.03	3.67 ± 0.02	3.65 ± 0.02
		*P*-value	<0.0001*	0.0001*	0.0001*
	COPD	Low	3.57 ± 0.03	3.47 ± 0.02	3.44 ± 0.02
		Moderate	3.70 ± 0.05	3.56 ± 0.04	3.53 ± 0.04
		High	4.00 ± 0.07	3.62 ± 0.05	3.59 ± 0.05
		*P*-value	<0.0001*	0.0195*	0.0220*
	*P*-value for interaction		0.3396	0.1053	0.1155
PEF	Normal	Low	7.31 ± 0.03	7.28 ± 0.03	7.26 ± 0.03
		Moderate	7.42 ± 0.04	7.31 ± 0.03	7.29 ± 0.03
		High	8.16 ± 0.07	7.40 ± 0.05	7.38 ± 0.05
		*P*-value	<0.0001*	0.0543	0.0592
	COPD	Low	6.02 ± 0.06	5.66 ± 0.05	5.64 ± 0.05
		Moderate	6.25 ± 0.09	5.76 ± 0.08	5.74 ± 0.08
		High	6.99 ± 0.17	5.98 ± 0.14	5.96 ± 0.14
		*P*-value	<0.0001*	0.0353*	0.0426*
	*P*-value for interaction		0.5598	0.3667	0.379

Values are presented as mean ± standard error (SE). The *p*-values were analyzed by analysis of covariance (ANCOVA). The *p*-value for interaction was estimated by including a cross-product term in the multivariable model. FEV1, forced expiratory volume in one second; FVC, forced vital capacity; PEF, peak expiratory flow; COPD, chronic obstructive pulmonary disease.

*P < 0.05.

† Not adjusted. ^‡^Adjusted for age, sex, body mass index (BMI), income, education, drinking, and smoking. ^§^ Adjusted for age, sex, BMI, income, education, drinking, smoking, diabetes mellitus, hypertension, and dyslipidemia.

### 3.3 Association between adherence to specific PA types and lung function

**FIGURE 1:**
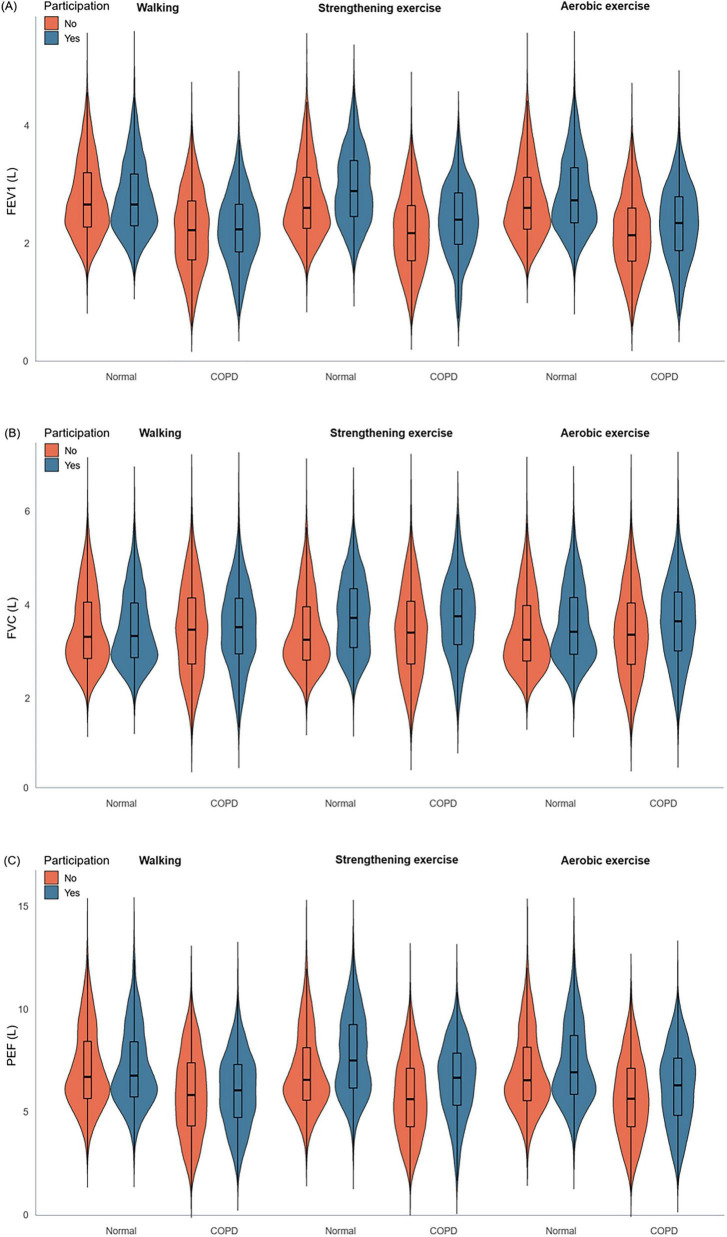
Violin plots showing the distributions of FEV1 **(A)**, FVC **(B)**, and PEF **(C)** according to adherence to walking, strengthening exercise, and aerobic exercise between the normal and COPD groups in Model 3. Adjusted for age, sex, body mass index (BMI), income, education, drinking, smoking, diabetes mellitus, hypertension, and dyslipidemia. FEV1, forced expiratory volume in one second; FVC, forced vital capacity; PEF, peak expiratory flow; COPD, chronic obstructive pulmonary disease.

Table 3 shows the association between adherence to three specific types of PA (walking, strengthening exercise, and aerobic exercise) and lung function parameters (FEV1, FVC, and PEF) in the normal and COPD groups. Walking participation was not significantly related to FEV1, FVC, or PEF in the COPD group. Only in the normal group, Model 2 and Model 3 revealed significant associations between walking participation and FEV1, FVC, and PEF (Table 3A). Strengthening exercise in the COPD group showed significant associations with FEV1 and FVC in Model 1, as well as with PEF in all models (Table 3B). Lastly, in the COPD group, adherence to aerobic exercise was significantly associated with increases in FEV1, FVC, and PEF across all models. Additionally, significant interaction effects were observed between COPD group and adherence to aerobic exercise for both FEV1 and FVC in Model 2 and 3 (FEV1: *p*-value for interaction = 0.0294 in Model 2 and 0.0297 in Model 3; FVC: *p*-value for interaction = 0.0067 in Model 2 and 0.0069 in Model 3). In other words, the increase in FEV1 and FVC associated with adherence to aerobic exercise was greater in the COPD group than in the normal group. Figure 1 presents violin plots illustrating the distributions of FEV1 (A), FVC (B), and PEF (C) according to adherence to walking, strengthening exercise, and aerobic exercise between the normal and COPD groups in Model 3.

**TABLE 3:** Association between lung function and adherence to walking, strengthening exercise, and aerobic exercise.

A. Walking					
Variables	Group	Walking	Model 1^†^	Model 2^‡^	Model 3^§^
FEV1	Normal	No	2.88 ± 0.01	2.81 ± 0.01	2.80 ± 0.01
		Yes	2.87 ± 0.01	2.83 ± 0.01	2.82 ± 0.01
		*P*-value	0.535	0.0221*	0.0204*
	COPD	No	2.34 ± 0.02	2.25 ± 0.02	2.24 ± 0.02
		Yes	2.34 ± 0.03	2.28 ± 0.02	2.26 ± 0.02
		*P*-value	0.9866	0.4908	0.4884
	*P*-value for interaction		0.8041	0.801	0.7938
FVC	Normal	No	3.64 ± 0.01	3.59 ± 0.01	3.57 ± 0.01
		Yes	3.62 ± 0.01	3.61 ± 0.01	3.59 ± 0.01
		*P*-value	0.5366	0.0226*	0.0203*
	COPD	No	3.64 ± 0.03	3.49 ± 0.02	3.47 ± 0.02
		Yes	3.65 ± 0.04	3.52 ± 0.03	3.50 ± 0.03
		*P*-value	0.8755	0.392	0.4139
	*P*-value for interaction		0.7186	0.8323	0.8306
PEF	Normal	No	7.42 ± 0.03	7.28 ± 0.03	7.26 ± 0.03
		Yes	7.42 ± 0.03	7.33 ± 0.03	7.31 ± 0.03
		*P*-value	0.9414	0.0177*	0.0169*
	COPD	No	6.15 ± 0.07	5.69 ± 0.06	5.67 ± 0.06
		Yes	6.20 ± 0.07	5.75 ± 0.06	5.74 ± 0.06
		*P*-value	0.6265	0.2172	0.6364
	*P*-value for interaction		0.673	0.9448	0.9088
**B. Strengthening exercise**					
**Variables**	**Group**	**Strengthening exercise**	**Model 1^†^**	**Model 2^‡^**	**Model 3^§^**
FEV1	Normal	No	2.83 ± 0.01	2.81 ± 0.01	2.80 ± 0.01
		Yes	3.05 ± 0.01	2.84 ± 0.01	2.83 ± 0.01
		*P*-value	<0.0001*	0.0891	0.1037
	COPD	No	2.29 ± 0.02	2.26 ± 0.02	2.25 ± 0.02
		Yes	2.48 ± 0.03	2.27 ± 0.03	2.26 ± 0.03
		*P*-value	<0.0001*	0.4956	0.4961
	*P*-value for interaction		0.3827	0.7378	0.7444
FVC	Normal	No	3.57 ± 0.01	3.60 ± 0.01	3.57 ± 0.01
		Yes	3.87 ± 0.02	3.63 ± 0.01	3.61 ± 0.01
		*P*-value	<0.0001*	0.0631	0.0774
	COPD	No	3.57 ± 0.03	3.49 ± 0.02	3.46 ± 0.02
		Yes	3.87 ± 0.05	3.56 ± 0.04	3.54 ± 0.04
		*P*-value	<0.0001*	0.1992	0.1959
	*P*-value for interaction		0.9786	0.2806	0.2755
PEF	Normal	No	7.26 ± 0.03	7.27 ± 0.02	7.25 ± 0.03
		Yes	8.04 ± 0.04	7.40 ± 0.03	7.38 ± 0.04
		*P*-value	<0.0001*	0.0007*	0.0008*
	COPD	No	5.98 ± 0.06	5.68 ± 0.05	5.66 ± 0.05
		Yes	6.73 ± 0.09	5.85 ± 0.08	5.83 ± 0.08
		*P*-value	<0.0001*	0.0364*	0.0395*
	*P*-value for interaction		0.8015	0.6218	0.6182
**C. Aerobic exercise**					
**Variables**	**Group**	**Aerobic exercise**	**Model 1^†^**	**Model 2^‡^**	**Model 3^§^**
FEV1	Normal	No	2.82 ± 0.01	2.81 ± 0.01	2.80 ± 0.01
		Yes	2.94 ± 0.01	2.83 ± 0.01	2.82 ± 0.01
		*P*-value	<0.0001*	0.0574	0.0769
	COPD	No	2.26 ± 0.02	2.23 ± 0.02	2.22 ± 0.02
		Yes	2.43 ± 0.03	2.30 ± 0.02	2.29 ± 0.02
		*P*-value	<0.0001*	0.0059*	0.0066*
	*P*-value for interaction		0.1871	0.0294*	0.0297*
FVC	Normal	No	3.56 ± 0.01	3.59 ± 0.01	3.57 ± 0.01
		Yes	3.72 ± 0.01	3.61 ± 0.01	3.59 ± 0.01
		*P*-value	<0.0001*	0.0131*	0.0204*
	COPD	No	3.54 ± 0.03	3.45 ± 0.02	3.43 ± 0.02
		Yes	3.78 ± 0.04	3.57 ± 0.03	3.55 ± 0.03
		*P*-value	<0.0001*	0.0051*	0.006*
	*P*-value for interaction		0.0886	0.0067*	0.0069*
PEF	Normal	No	7.24 ± 0.03	7.26 ± 0.03	7.24 ± 0.03
		Yes	7.63 ± 0.03	7.34 ± 0.03	7.33 ± 0.03
		*P*-value	<0.0001*	0.0012*	0.0015*
	COPD	No	5.95 ± 0.07	5.63 ± 0.05	5.61 ± 0.06
		Yes	6.45 ± 0.08	5.82 ± 0.06	5.80 ± 0.07
		*P*-value	<0.0001*	0.0182*	0.0199*
	*P*-value for interaction		0.2638	0.1884	0.1849

Values are presented as mean ± standard error (SE). The *p*-values were analyzed by ANCOVA. The *p*-value for interaction was estimated by including a cross-product term in the multivariable model. FEV1, forced expiratory volume in one second; FVC, forced vital capacity; PEF, peak expiratory flow; COPD, chronic obstructive pulmonary disease.

*P < 0.05.

† Not adjusted. ^‡^Adjusted for age, sex, body mass index (BMI), income, education, drinking, and smoking. ^§^ Adjusted for age, sex, BMI, income, education, drinking, smoking, diabetes mellitus, hypertension, and dyslipidemia.

### 3.4 Association of intensity level of physical activity with lung function in the normal and COPD groups according to age and sex

**FIGURE 2:**
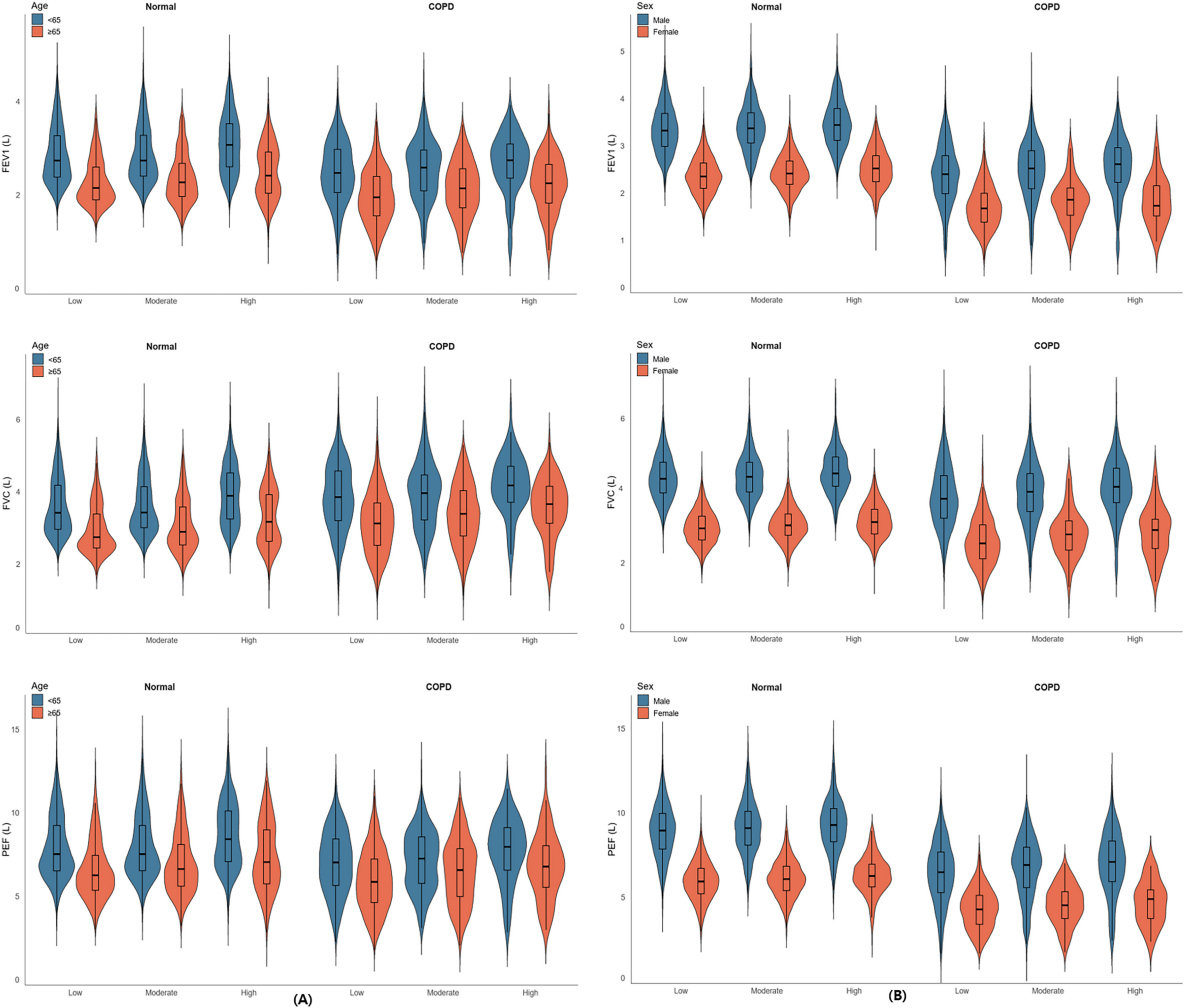
Violin plots of FEV1, FVC, and PEF by physical activity intensity level in the normal and COPD groups, stratified by age **(A)** and sex **(B)** in Model 3. Adjusted for age, sex, body mass index (BMI), income, education, drinking, smoking, diabetes mellitus, hypertension, and dyslipidemia. FEV1, forced expiratory volume in one second; FVC, forced vital capacity; PEF, peak expiratory flow; COPD, chronic obstructive pulmonary disease. This figure is complemented with a Supplementary Table 1 providing detailed descriptions of the corresponding results.

Figure 2 shows violin plots of FEV1, FVC, and PEF by physical activity intensity level in the normal and COPD groups, stratified by age (A) and sex (B) in Model 3. In participants younger than 65 years, only the normal group showed significant associations between PA intensity level and FEV1 and FVC (Supplementary Table 1A). In females, only the normal group showed significant associations between PA intensity level and FEV1 and FVC; in males, the normal group revealed significant associations between PA intensity level and only FVC (Supplementary Table 1B). No significant associations between PA intensity level and lung function were observed in the COPD group when stratified by age or sex.

## 4 Discussion

This study demonstrated that higher PA intensity was significantly associated with improved FEV1, FVC, and PEF in COPD patients in a dose-response manner, even after full covariate adjustment. Among the three PA types examined, aerobic exercise was the only type significantly associated with all three lung function parameters in COPD patients, and its associations with FEV1 and FVC were significantly stronger in the COPD group than in individuals with normal lung function, a finding that, to our knowledge, has not been previously demonstrated at the population level. Furthermore, when COPD patients were stratified by age or sex, the association between PA intensity and lung function was no longer significant, suggesting that the type of PA, rather than intensity alone, may be the more critical determinant of pulmonary benefit in this population. Collectively, these findings underscore the importance of incorporating structured aerobic exercise as a core component of comprehensive COPD management.

Several studies have highlighted the importance of PA in patients with COPD. A systematic review of 86 studies showed that PA was consistently associated with hyperinflation, exercise capacity, dyspnea, previous exacerbations, gas exchange, systemic inflammation, quality of life, and self-efficacy, with mortality and exacerbations showing consistent associations based on moderate levels of evidence (17). Despite this well-established role, few studies have investigated the specific relationship between PA intensity and lung function in COPD patients, and existing evidence has been inconsistent. Han et al. in the CODA study involving 504 participants, reported a significant association between PA and FEV1 only in the mild COPD subgroup (9), while Pitta et al. in a study of 40 COPD patients, found that maximal voluntary ventilation was associated with PA intensity but FEV1 was not (5). Our findings extend this evidence by demonstrating a significant dose-response association between PA intensity and FEV1, FVC, and PEF in COPD patients across all adjustment models, suggesting that higher PA intensity confers a measurable pulmonary benefit even in the presence of established disease.

We investigated the association between lung function and PA by categorizing activity into walking, strengthening exercise, and aerobic exercise. Aerobic exercise was the only type significantly associated with improved FEV1, FVC, and PEF in COPD patients after full covariate adjustment, while walking and strengthening exercise showed no significant associations with lung function in the COPD group. Notably, the associations between aerobic exercise adherence and FEV1 and FVC were significantly stronger in the COPD group than in the normal group (*p* for interaction = 0.0297 and 0.0069, respectively), whereas no such differential effect was observed for PEF. These findings are consistent with prior evidence demonstrating that aerobic exercise improves lung function, including FEV1 and FVC, in patients with COPD (18, 19).

Pulmonary rehabilitation typically includes endurance training, which corresponds to aerobic exercise in our study, and strengthening exercises, and may also involve respiratory muscle training and other modalities tailored to the patient’s clinical condition. These programs have been shown to improve exercise capacity, quality of life, and dyspnea in patients with COPD (20–22). In particular, the superior association between aerobic exercise and lung function observed in our study may be explained by several physiological mechanisms inherent to endurance training in pulmonary rehabilitation. First, repeated endurance loading enhances the oxidative capacity of respiratory and peripheral muscles, reducing ventilatory demand at a given workload and improving ventilatory efficiency (23). Second, aerobic training mitigates dynamic hyperinflation, thereby reducing exertional dyspnea and improving heart rate recovery (24, 25). Third, skeletal muscle dysfunction in COPD, characterized by impaired aerobic metabolism and premature lactic acidosis, suggests that exercise intolerance stems not only from respiratory impairment but also from peripheral limitations (26–28). Regular aerobic exercise addresses these extrapulmonary components through systemic anti-inflammatory effects; contracting skeletal muscles release IL-6 as a myokine, which suppresses TNF-α and promotes anti-inflammatory cytokine production, potentially contributing to preservation of lung function (29). Taken together, these adaptations provide a biologically plausible explanation for the preferential pulmonary benefits of aerobic exercise in COPD patients observed in this study.

Although this study did not demonstrate a significant association between walking and lung function in COPD patients, prior studies have reported that walking-based training improves quality of life and exercise capacity in this population (30, 31). Furthermore, the clinical limitations commonly experienced by COPD patients, such as severe exertional dyspnea and fear of breathlessness, underscore the potential role of walking as a practical entry point for endurance training in pulmonary rehabilitation. However, as exercise tolerance improves, a gradual progression toward higher-intensity aerobic exercise should be considered, given our finding that aerobic exercise, rather than walking alone, was significantly associated with improved lung function in COPD.

Unlike aerobic exercise, strengthening exercise was not significantly associated with FEV1 or FVC in COPD patients after full covariate adjustment, with only a marginal association observed for PEF. Nevertheless, limb muscle dysfunction is a major systemic manifestation of COPD, and limb muscle atrophy and weakness contribute to reduced endurance, increased fatigue, and even premature mortality (32–34). Notably, COPD patients in our study demonstrated higher participation in strengthening exercise than the normal group (25.8% vs. 21.3%, *p* < 0.0001). Although formal pulmonary rehabilitation participation rates remain relatively low in Korea, this finding may reflect growing clinical awareness of sarcopenia and limb muscle dysfunction as systemic manifestations of COPD among Korean pulmonologists, which may lead to more frequent physician recommendations for strengthening exercise in this population, even outside of structured rehabilitation programs. While our study did not demonstrate a significant association between strengthening exercise and lung function, prior studies have shown positive effects of strengthening interventions on quality of life, muscle condition, and functional performance in COPD patients (35, 36). Moreover, combining strengthening exercises with aerobic exercise has been shown to result in greater improvements in muscle strength and size (37). Given these findings, strengthening exercise remains an important component of comprehensive COPD management alongside aerobic exercise, even though its direct contribution to lung function appears limited.

We further examined the association between PA intensity and lung function in COPD patients stratified by age and sex. No significant associations were observed in any subgroup, suggesting that the effect of PA intensity on lung function in COPD patients is not uniform across demographic groups. This finding is consistent with our overall interpretation that PA type, specifically aerobic exercise, may be a more critical determinant of pulmonary benefit in COPD than intensity alone, regardless of age or sex. Future studies incorporating objective PA measurement and longitudinal designs are needed to clarify whether targeted aerobic exercise interventions yield differential benefits across these subgroups.

This study has several limitations. First, the cross-sectional design based on KNHANES data prevents establishment of causality between PA intensity or type and lung function. Reverse causality cannot be excluded, as better lung function may lead to higher levels of PA. Future longitudinal studies and randomized controlled trials are warranted to clarify these causal relationships. Second, selection bias cannot be excluded, as COPD patients enrolled through structured health surveys may represent relatively healthier individuals more engaged with their health. Third, COPD severity could not be classified according to GOLD criteria, as the KNHANES dataset identified COPD based on the FEV1/FVC ratio < 0.7 without information on predicted FEV1%. Residual confounding by disease severity, which is an important determinant of both PA and lung function, may therefore remain. Additionally, because COPD was defined using the FEV1/FVC ratio alone, patients with asthma may have been misclassified as having COPD. Fourth, PA was assessed by self-report using the GPAQ, which is subject to recall bias; however, the GPAQ has been validated as a reliable tool for PA assessment in prior studies. Finally, as all participants were Korean, generalizability to other ethnic groups may be limited, given that COPD prevalence and the impact of risk factors vary considerably across populations (4).

Despite these limitations, this study has several notable strengths. First, the large, nationally representative sample of 14,679 participants drawn from KNHANES enhances the generalizability and robustness of our findings. Second, three adjustment models with progressively increasing covariate control, including sociodemographic factors, anthropometric measures, and major comorbidities, were applied to minimize residual confounding. Third, by categorizing PA into three distinct types and formally testing whether their associations with lung function differed between COPD and normal groups, this study provides the first population-level evidence that aerobic exercise is more strongly associated with lung function in COPD patients than in individuals with normal lung function. Finally, stratified analyses by age and sex further characterized the conditions under which PA intensity confers pulmonary benefit in COPD, adding demographic nuance to the findings.

## 5 Conclusion

Higher PA intensity was significantly associated with improved lung function in COPD patients in a dose-response manner, though this association varied by subgroup. Additionally, among the PA types examined, aerobic exercise was the only type significantly associated with improved FEV1, FVC, and PEF in COPD patients after full covariate adjustment; moreover, the associations with FEV1 and FVC were significantly stronger in the COPD group than in individuals with normal lung function. Subgroup analyses further revealed that the association between PA intensity and lung function was not significant in COPD patients when stratified by age or sex, underscoring that the type of PA, rather than intensity alone, may be the more critical determinant of pulmonary benefit in COPD. These findings support the inclusion of structured aerobic exercise as a core component of comprehensive COPD management.

## Data Availability

The datasets presented in this study can be found in online repositories. The names of the repository/repositories and accession number(s) can be found below: the datasets generated and/or analyzed during the current study (KNHANES 2014-2019) are available in the Korea National Health and Nutrition Examination Survey (KNHANES) repository maintained by the Korea Disease Control and Prevention Agency (KDCA), https://knhanes.kdca.go.kr.
