# Supplementary material for: Association between intensity and type of physical activity and lung function in patients with COPD: a population-based study
Source: Front Med (Lausanne). 2026 Jul 24;13:1878202. doi: 10.3389/fmed.2026.1878202 (PMC13447420; doi:10.3389/fmed.2026.1878202)

Supplementary Material

Table S1. Associations between the level of physical activity and lung function parameters (FEV1, FVC, and PEF) in the normal and COPD groups, stratified by age (A) and sex (B), in Model 3.

(A) Age

Subgroup	Group	Physical activity level	FEV1 Model 3	FVC Model 3	PEF Model 3
**Age <65**	Normal	Low	2.94±0.01	3.71±0.01	7.50±0.04
		Moderate	2.94±0.01	3.72±0.01	7.53±0.04
		High	2.99±0.01	3.79±0.02	7.62±0.06
		***p*-value**	0.0037*	0.0002*	0.0752
	COPD	Low	2.37±0.03	3.66±0.04	5.82±0.08
		Moderate	2.47±0.04	3.78±0.05	5.94±0.12
		High	2.46±0.05	3.82±0.07	6.26±0.19
		***p*-value**	0.0753	0.0534	0.0983
	***p*-value for interaction**		0.1643	0.1848	0.2702
**Age ≥65**	Normal	Low	2.40±0.02	3.17±0.02	6.50±0.06
		Moderate	2.42±0.02	3.19±0.02	6.57±0.08
		High	2.38±0.03	3.17±0.04	6.43±0.13
		***p*-value**	0.2981	0.3737	0.3395
	COPD	Low	1.86±0.02	2.98±0.03	5.13±0.07
		Moderate	1.91±0.03	3.04±0.04	5.20±0.10
		High	1.88±0.06	3.06±0.08	5.23±0.19
		***p*-value**	0.2892	0.4195	0.6636
	***p*-value for interaction**		0.6844	0.5364	0.7676

(B) Sex

Subgroup	Group	Physical activity level	FEV1 Model 3	FVC Model 3	PEF Model 3
**Male**	Normal	Low	3.23±0.01	3.72±0.01	8.63±0.04
		Moderate	3.22±0.01	3.74±0.01	8.64±0.05
		High	3.27±0.02	3.87±0.02	8.72±0.08
		***p*-value**	0.0623	0.0024*	0.3371
	COPD	Low	2.62±0.02	3.71±0.03	6.87±0.07
		Moderate	2.69±0.03	3.84±0.04	6.96±0.10
		High	2.70±0.05	3.92±0.06	7.25±0.16
		***p*-value**	0.1059	0.118	0.0895
	***p*-value for interaction**		0.1643	0.1848	0.2702
**Female**	Normal	Low	2.37±0.01	4.15±0.02	5.82±0.04
		Moderate	2.39±0.02	4.12±0.02	5.85±0.05
		High	2.41±0.02	4.22±0.03	5.89±0.07
		***p*-value**	0.0096*	<.0001*	0.1835
	COPD	Low	1.93±0.03	4.03±0.03	4.63±0.07
		Moderate	1.99±0.04	4.11±0.04	4.76±0.09
		High	2.06±0.12	4.16±0.06	4.99±0.31
		***p*-value**	0.2533	0.0701	0.4482
	***p*-value for interaction**		0.6844	0.5364	0.7676

Values are presented as mean±standard error (SE).
The *p*-values were analyzed by ANCOVA.
* $p < 0.05$.
Adjusted for age, sex, BMI, income, education, drinking, smoking, diabetes mellitus, hypertension, and dyslipidemia.
The *p*-value for interaction was estimated by including a cross-product term in the multivariable model.
FEV1, forced expiratory volume in one second; FVC, forced vital capacity; PEF, peak expiratory flow; COPD, chronic obstructive pulmonary disease

Table S2. Inclusion and exclusion criteria for study population

Inclusion criteria	Exclusion criteria
Aged ≥40 years	Aged <40 years
Completed spirometry	Missing spirometry data
Obstructive or normal spirometry pattern (FEV1/FVC <0.7, or FEV1/FVC ≥0.7 and FVC ≥80%)	Restrictive ventilatory pattern (FVC <80% and FEV1/FVC ≥0.7)
Completed physical activity questionnaire (GPAQ)	Missing physical activity data
Complete data on all covariates (sociodemographic factors, anthropometric measures, and comorbidities)	Missing covariate data
**Group classification**	
COPD group: FEV1/FVC <0.7 (n = 2,293)	
Normal spirometry group: FEV1/FVC ≥0.7 and FVC ≥80% (n = 12,386)	

Figure S1. Flowchart for study population from the Korea National Health and Nutrition Examination Survey (KNHANES) 2014–2019.

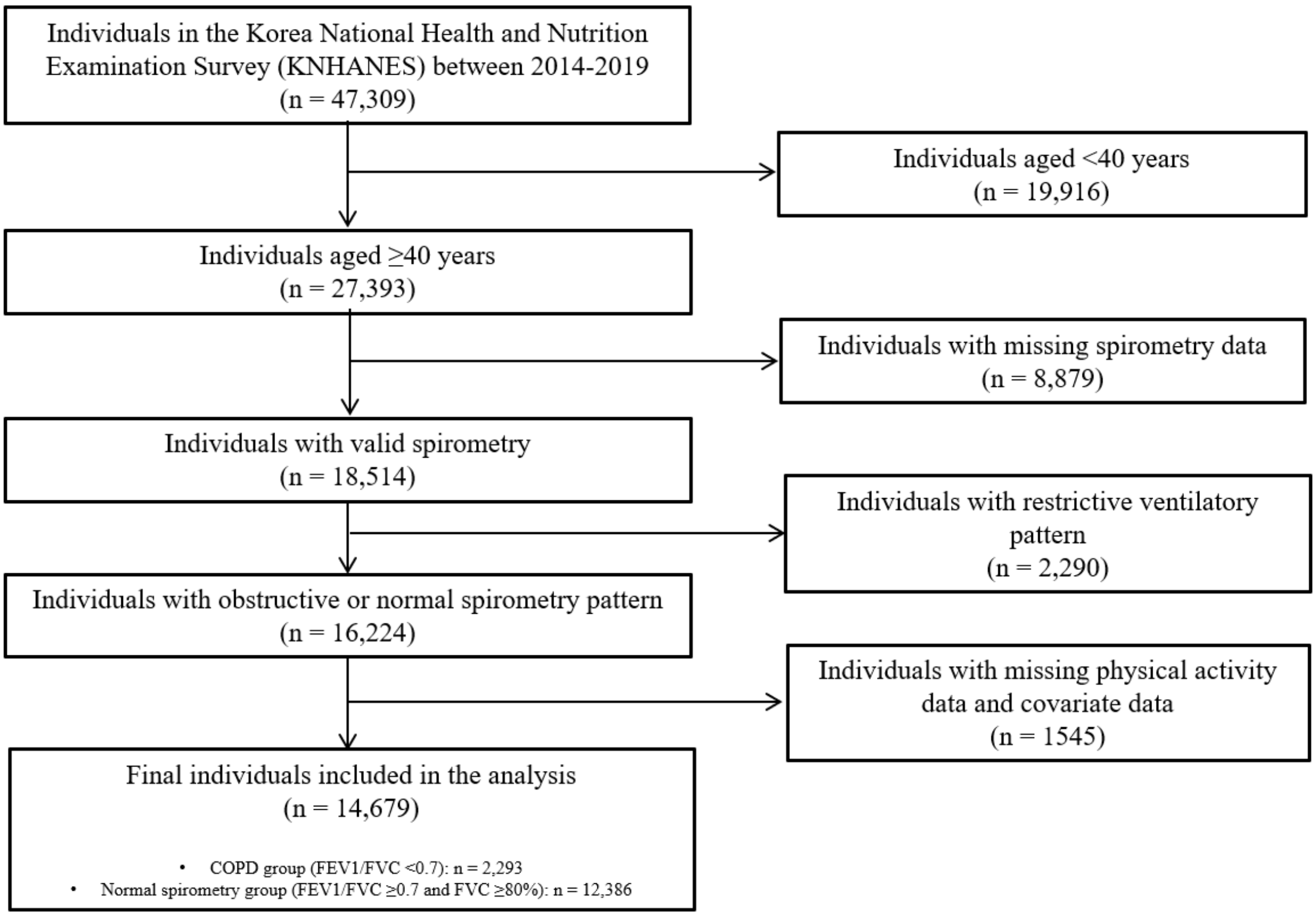

Supplement: Supplementary file 1 [file Data_Sheet_1.pdf]
